# Navigation strategies in *Caenorhabditis elegans* are differentially altered by learning

**DOI:** 10.1371/journal.pbio.3003005

**Published:** 2025-03-21

**Authors:** Kevin S. Chen, Anuj K. Sharma, Jonathan W. Pillow, Andrew M. Leifer

**Affiliations:** 1 Princeton Neuroscience Institute, Princeton University, Princeton, New Jersey, United States of America; 2 Department of Physics, Princeton University, Princeton, New Jersey, United States of America; Brandeis University, UNITED STATES OF AMERICA

## Abstract

Learned olfactory-guided navigation is a powerful platform for studying how a brain generates goal-directed behaviors. However, the quantitative changes that occur in sensorimotor transformations and the underlying neural circuit substrates to generate such learning-dependent navigation is still unclear. Here we investigate learned sensorimotor processing for navigation in the nematode *Caenorhabditis elegans* by measuring and modeling experience-dependent odor and salt chemotaxis. We then explore the neural basis of learned odor navigation through perturbation experiments. We develop a novel statistical model to characterize how the worm employs two behavioral strategies: a biased random walk and weathervaning. We infer weights on these strategies and characterize sensorimotor kernels that govern them by fitting our model to the worm’s time-varying navigation trajectories and precise sensory experiences. After olfactory learning, the fitted odor kernels reflect how appetitive and aversive trained worms up- and down-regulate both strategies, respectively. The model predicts an animal’s past olfactory learning experience with  > 90*%* accuracy given finite observations, outperforming a classical chemotaxis metric. The model trained on natural odors further predicts the animals’ learning-dependent response to optogenetically induced odor perception. Our measurements and model show that behavioral variability is altered by learning—trained worms exhibit less variable navigation than naive ones. Genetically disrupting individual interneuron classes downstream of an odor-sensing neuron reveals that learned navigation strategies are distributed in the network. Together, we present a flexible navigation algorithm that is supported by distributed neural computation in a compact brain.

## Author summary

Learning is a feature of species across scales. How does the brain flexibly learn to produce complex behavior? We focus on *C. elegans* to study how navigation strategies depend on olfactory learning by utilizing precise measurements and a novel model. The fitted model shows that learning alters two known navigation strategies in worms and the model outperforms a classical metric in decoding the animal’s past learning experience. We discover that learned navigation can express itself in a context-dependent manner. In addition, through perturbing individual interneurons, we find that most neurons’ contributions are distributed across strategies and are differentially altered by learning. We expect these flexible behavioral algorithms and neural computations to be generalized to other species and behavior.

## Abstract 3

How do worms navigate based on learned experience? This study presents a statistical model and in vivo experimental data to reveal flexible behavioral strategies and distributed neural computations that underlie learning-dependent odor navigation in Caenorhabditis elegans.

## Introduction

Learning is a fundamental property of neural systems that allows an animal to flexibly alter behavior based on experience. Learned olfactory navigation provides an ideal framework for studying learning because it is a naturalistic behavior, common to species across scales [1,2], ethologically relevant for seeking food [3,4] and avoiding pathogens [5,6], and can be rapidly learned in very few trials [4,7,8]. Careful characterization of learned navigation behavior in a naturalistic context can shed light on the flexible computation performed by the brain [9].

To study how animals flexibly alter their olfactory navigation upon learning, we focus on the nematode worm *C. elegans*. This worm has a compact nervous system [10], well-characterized olfactory neural circuits [11–15] and navigation behavior that has been studied in detail [11,16]. Worms learn to associate an odor with the presence or absence of food and then will either navigate towards higher concentrations of the odor or will ignore the odor, respectively [3,17]. When learning to associate the odor butanone to a food source, it is unknown how the worm’s navigation strategies are altered by learning. We aim to quantitatively characterize the worm’s navigation strategy and the underlying sensory transformations in order to constrain neural mechanisms of learned navigation.

The worm navigates in sensory environments mainly through two strategies: klinotaxis and klinokinesis [16,18,19]. Klinotaxis is a process in which the worm continuously modulates its heading to align with the local gradient in space, also known as “weathervaning” [18]. Klinokinesis is a biased random walk [20,21] in which the worm produces sharp turns called “pirouettes” with a probability that depends on the animal’s estimate of the local gradient of a sensory cue [16,20,22]. In both cases the animal estimates information about the local gradient by comparing measurements of the stimuli across time and space: during klinokinesis the worm samples odor concentration at different points along its locomotory trajectory, while for klinotaxis it samples concentrations between shorter side-to-side swings of its head. Both strategies contribute to sensory-guided navigation in landscapes of temperature [19], salt [18,23–25], and certain odors [11,18,26,27].

Experience-dependent changes to these behavioral strategies have been characterized in some sensory modalities, such as in response to salt [18,23–25], temperature [19,28,29], and bacterial food [6,30,31], but it remains less known for learning in response to odorants, such as butanone. For instance, it is not known whether worms adopt entirely new navigation strategies after odor learning, or whether they modulate existing ones. It is plausible that olfactory learning could influence behavioral strategies in a manner similar to learning in the context of salt and temperature, but this hypothesis still requires empirical validation.

Quantitative analysis for airborne odor-guided navigation has been limited, in part because of experimental challenges in measuring detailed information about the odor concentration experienced by the animal. While past work estimated odor cues indirectly or with models [4,18], precise concentration measurements are required to characterize sensorimotor transformation in the sensory environment. Recently, however, new experimental methods to control and monitor the odor landscape experienced by small animals, such as the worm, [32–34], now make it possible to empirically constrain quantitative models of odor-guided navigation, as we pursue here.

Worms are intrinsically attracted to butanone, a volatile organic compound found in bacterial food in the worm’s natural habitat [35]. When worms are exposed to butanone paired with food (“appetitive training”), they increase their attraction and are more likely to navigate towards higher butanone concentrations [3,12,17,36]. In contrast, when butanone is paired with starvation (“aversive training”), worms decrease their tendency to climb up butanone gradients in comparison to worms without exposure to butanone (“naive”) [3,36]. The animal’s neural and behavioral responses to butanone both change upon learning [12,36]. Sensory neuron AWC^ON^ responds to butanone [3,36], as well as others [12,37]. However, the involvement of downstream interneurons in butanone learning and learned navigation are still unclear.

In this study we seek to answer: (1) How are the worm’s navigation strategies altered by olfactory learning? (2) How are sensorimotor transformations altered by learning and how does this vary across a population? (3) What neural substrates may be involved? To answer these questions, we combine precise experimental measurements using our recently developed continuous odor monitoring assay [32] and a novel statistical model to rigorously characterize how butanone associative learning alters odor navigation strategies in worms.

Our measurements and model reveals that the animal’s biased random walk is differentially altered by butanone learning and that its weathervaning strategy is down-regulated upon aversive learning. Our approach yields interpretable model parameters that better decode training conditions compared to chemotaxis index, and also predict response to optogenetic perturbation in the sensory neuron AWC^ON^. We discover that naive worms have higher behavioral variability and demonstrate context-dependent behavior. And we provide insights into the role of specific interneurons.

## Results

### Learning differentially alters olfactory navigation

**Fig 1:**
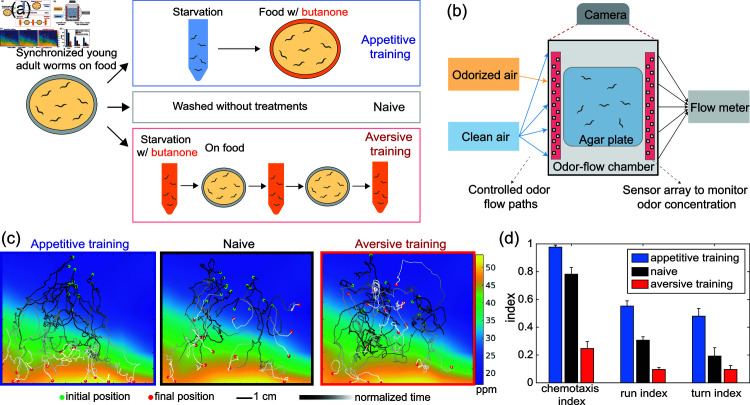
Differentially modulated olfactory learning in *C. elegans.* (a) Protocol for butanone associative training in worms. (b) After exposure to different training regimens in (a), worms’ olfactory navigation is measured in a controlled odor environment. (c) Example trajectory after three different training conditions. (d) Summary statistics of learning across three conditions. Error bar shows standard error of mean across 9-13 plates for each condition. For each index, all pairs of training conditions (appetitive-naive, naive-aversive, and appetitive-aversive) show statistically significant differences (t-test, *p* < 0 . 05), except for the turn index when comparing naive to aversive conditions. All data underlying this figure can be found at doi: 10.6084/m9.figshare.24764403.

We developed a protocol to train worms to associate butanone with either food (appetitive training) or starvation (aversive training) (Fig 1a). Our protocol was similar to previously reported training regimens [12,17,36] except that ours exposes the animal to multiple rounds of odor paired with starvation instead of only one, which we found increases consistency in learning (Fig A in S1 File). After training, we recorded the movement of populations of worms as they crawled in a defined odor landscape that used metal-oxide sensors to continuously monitor the odor concentration along the boundary [32] (Fig 1b; Fig B in S1 File). We recorded hundreds of locomotory trajectories per plate in this odor environment after different training conditions, for up to 13 plates per training condition. Animal’s locomotory trajectories were qualitatively different depending upon learning, with appetitive trained animals traveling up-gradient more often than naive animals, and aversive-trained animals traveling up-gradient least of all, broadly consistent with prior reports [3,12,36] (Fig 1c). We quantify the performance of traveling up the gradient using a “chemotaxis index”, $\frac{N_{u}-N_{d}}{N_{u}+N_{d}}$, that compares the number of trajectories going up-gradient, $N_{u}$, to the number of trajectories going down-gradient $N_{d}$ [3,36] (Fig 1d). Performance navigating up-gradients is differentially modulated by two training paradigms: chemotaxis index increases after appetitive training and decreases after aversive training compared to naive worms that undergo no training. Interestingly, even animals that undergo aversive training do not, on average, navigate down the gradient. This suggests that after learning an association between butanone and starvation, animals are indifferent or at most only still slightly attracted to butanone.

To explore whether worm navigation superficially resembles a biased random walk, we calculated a “run index”, $\frac{r_{u}-r_{d}}{r_{u}+r_{d}}$, by computing the normalized length of a run moving up-gradient, where the run *r* is defined as the period between pirouettes, and subscripts _*u*_ and _*d*_ denote up- or down-gradient (Fig 1d). Similarly, to explore whether navigation superficially resembles a weathervaning strategy, we calculated a “turn index”, $\frac{p_{u}-p_{d}}{p_{u}+p_{d}}$, that reports the fraction of turn events *p* that result in heading up-gradient to those heading down-gradient [23]. Additional details are described in the Methods section.

While the metrics above suggest hypotheses about how navigational strategies change due to learning, they provide little information about the dynamics of navigation, nor the sensorimotor transformations that govern these dynamics. Specifically, the metrics use only binary information about whether at the end of their track the animal traveled up- or down-gradient, and it ignore details of the sensory landscape in between and omits dynamic information like the odor concentration the animal experienced over time. The indices also provide no information about the behavioral variability across the population. To overcome these limitations, we sought a statistical model that captures temporal information, behavioral noise, and explicitly predicts how the animal changes its movement in response to sensory stimuli.

### Odor-dependent mixture model of olfactory navigation

**Fig 2:**
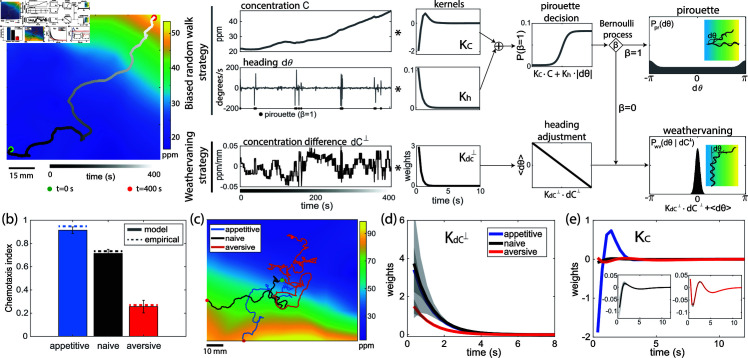
dPAW captures learned olfactory navigation in worms. (a) Schematic of the dPAW model. An example measured trajectory is shown on the far left, providing time series of concentration and angle changes. Response kernels and decision functions are fitted to the time series data. The Bernoulli process *β* leads to two parallel strategies: biased random walk and weathervaning. (b) Chemotaxis index of simulated trajectories with inferred parameters. Error bar shows standard error of mean across 10 repeated simulation, each with 100 trajectories. (c) Example trajectories simulated from each training conditions. (d) Kernel $K_{dC^{⊥}}$ and (e) Kernel $K_{C}$ fitted to three training conditions. Shaded area shows standard deviation of the kernel estimate. All data underlying this figure can be found at doi: 10.6084/m9.figshare.24764403.

To characterize how olfactory sensory inputs are transformed to behavior under different training conditions, we developed a dynamic Pirouette and Weathervaning (dPAW) statistical model of worm olfactory navigation. The dPAW model consists of a mixture of two navigation strategies: a pirouette behavior consisting of an abrupt change in heading angle (a turn) and weathervaning, which instead continuously modulates heading angle. The dPAW model describes how the worm implements and balances these two behavioral strategies depending on time-varying sensory inputs (Fig 2a). The dPAW model is an extension to a classic biased random walk, where the “run” intervals are replaced with weathervaning behavior. This framework explicitly models these strategies and is fit to detailed measurements of movement and odor-experience. This is to our knowledge the first statistical model that explicitly captures the detailed changes to *C. elegans* navigation strategies upon butanone learning.

The model worm samples its heading change *dθ* at each time step from one of two distributions: either a “weathervaning distribution” $P_{wv}(d\theta )$ or a “pirouette distribution” $P_{pr}(d\theta )$. The weathervaning distribution is narrow, reflecting small changes in heading angle that result from the worm’s recent measurements of the concentration gradient. Pirouette behavior, on the other hand, corresponds to large turns that the worm makes when it receives evidence that it is going in the wrong direction. The pirouette distribution is therefore broad, with a peak at  ± *π*, indicating a complete reversal in direction. The concentration-dependent “decision” to produce a pirouette in between runs corresponds to a classic model known as “biased random walk” [16,22].

The worm’s decision to initiate a turn (as part of a pirouette) or to continue to weathervane on each time step is modeled with a Bernoulli generalized linear model (GLM) that takes the filtered history of the odor concentration $C_{1:t-1}$ and the worm’s own movement history $d\theta_{1:t-1}$ as inputs. The output of this GLM is a binary variable $\beta_{t}$ that indicates the presence of a pirouette. Thus, the worm samples its heading change from the pirouette distribution $P_{pr}(d\theta )$ if $\beta_{t}=1$ and the weathervaning distribution $P_{wv}(d\theta )$ if $\beta_{t}=0$. The full model can be written:


$$\begin{array}{ccc} P(d\theta_{t}|C_{1:t-1},dC_{1:t-1}^{⊥},d\theta_{1:t-1})=P(\beta_{t}=1)P_{pr}(d\theta )+P(\beta_{t}=0)P_{wv}(d\theta ), & & \end{array}$$ (1)

where


$$\begin{array}{ccc} P(\beta_{t}=1)=m+\frac{M-m}{1+\exp(K_{C}\cdot C_{1:t-1}+K_{h}\cdot |d\theta_{1:t-1}|)} & & \end{array}$$ (2)

is the mixing probability over the two distributions, with parameters *m* and *M* for the minimum and maximum probability of a pirouette on a single time bin, and $K_{C}$ and $K_{h}$ corresponding to filters on past odor concentration $C_{1:t-1}$ and the past absolute angular change $\left|d\theta_{1:t-1}\right|$ vectors, respectively. The pirouette and weathervaning distributions are in turn given by


$$\begin{array}{ccc} P_{pr}(d\theta ) & =\alpha U[-\pi ,\pi ]+(1-\alpha )f(\pi ,\kappa_{pr}), & \end{array}$$ (3)

$$\begin{array}{ccc} P_{wv}(d\theta |dC_{1:t-1}^{⊥}) & =f(-K_{dC^{⊥}}\cdot {dC}_{1:t-1}^{⊥},\kappa_{wv}) & \end{array}$$ (4)

where *U* is uniform in the circular heading and *f* is a von Mises distribution with mean and precision parameter *κ*. In the pirouette distribution, scalar *α* ∈ [ 0 , 1 ]  is the weight on the uniform distribution and $\kappa_{pr}$ is the precision parameter that determines the sharpness of the pirouette. In the weathervaning distribution, the mean is altered according to the perpendicular concentration change $dC_{1:t-1}^{⊥}$ and the precision parameter $\kappa_{wv}$ determines the noise around the head angle. (Note we have made a simplification by allowing the model access to the instantaneous perpendicular odor concentration. In reality, the animal is thought to compute $dC_{1:t-1}^{⊥}$ from sequential measurements in time as it swings its head through space.) The full dPAW model has 13 parameters, which includes all the mixture parameters in Eqs 2–4 and all parameters needed to prescribe the shape of the kernels. Model setting and inference is described in the Methods section.

We fit dPAW to measurements of animal movement in the odor arena, including the time varying headings $d\theta_{t}$, the measured odor concentrations $C_{t}$ along the worm’s locomotion path, and the concentration perpendicular to the locomotion path $dC_{t}^{⊥}$. All model parameters in dPAW are jointly inferred through a maximum-likelihood method. To validate that parameters are reliably inferred, we simulated example chemotaxis trajectories from pre-defined parameters, fit them by the model, and confirmed that the model accurately recovered the pre-defined parameters (Fig C in S1 File). In the rest of the paper we fit the model to measurements to explore how navigation strategies change with learning.

### Model captures navigation altered by olfactory learning

To characterize how olfactory learning alters navigation strategies, we fit the dPAW model to trajectories measured in the butanone odor environment after different training conditions. We first confirmed that the model captures key aspects of the animal’s navigation. We confirmed that the fitted model’s estimate of pirouette frequency matches that measured empirically [16] (Fig Db in S1 File). We also used the model to simulate chemotaxis behavior in the odor environment and confirmed that model-generated trajectories have a chemotaxis index that agrees with measurement and is similarly differentially modulated by training conditions (Fig 2b). Model-generated trajectories also appear visually similar to experimental observations (Fig 2c). And in further agreement, trajectories simulated from the model recapitulate sensory and behavioral statistics measured in experiments, including the measured distribution of the worm’s heading angle, pirouette rate, experienced perpendicular odor concentration difference, and tangential odor concentration (Fig D and Fig E in S1 File).

Agreement between model and measurement was not due to chance. The dPAW model on average captures 0 . 3 to 0 . 6 bits/s more information about navigation behavior than a null model that lacks any olfactory sensing mechanisms (Fig 4c). For comparison to additional null models see Fig E in S1 File.

### Sensorimotor kernels change upon learning

We hypothesize that the sensorimotor temporal kernels, $K_{C}$ and $K_{dc^{⊥}}$, should change upon learning [16,18,36]. An alternative hypothesis is that changes to baseline pirouette rate and trajectory curvature alter the chemotaxis index without having to change the kernel governing its sensorimotor response. To test this, we inspected the inferred kernels fit to animals that had underwent different training conditions. Kernels corresponding to both weathervaning and biased random walk strategies were altered by learning (Fig 2d and 2e). The weathervaning kernel $K_{dC^{⊥}}$ had lower weights after aversive training compared to appetitive trained or naive animals (Fig 2d), suggesting that the animal’s heading angle is less tightly dependent upon the concentration difference perpendicular to its path. In other words, aversive trained worms don’t pay as much attention to perpendicular concentration when choosing their heading angle. There are two extreme ways these worms could not pay attention to the sensory cue: the worm could be uncoordinated and randomly select a heading direction, or alternatively, it could keep its existing heading. We observe the latter. We found that aversive-trained worms decrease their behavioral noise and are more likely to preserve their existing heading and exhibit higher persistent length during their runs, as indicated by higher fitted precision parameter of weathervaning $\kappa_{wv}$ (Fig 4b).

The kernels corresponding to pirouette decisions, $K_{C}$, change for both aversive and appetitive training compared to naive animals (Fig 2e). The amplitude is increased after appetitive training compared to naive animals, suggesting that appetitive trained animal’s pirouette probability depends more strongly on the concentration change along the navigation path than for naive animals. After aversive training, the kernel $K_{C}$ has a longer time delay and forms a tri-phasic shape, markedly different from the biphasic shape observed in naive animals, suggesting that aversive trained animals may be less responsive to downward changes in concentration (Fig 2e).

Collectively, we show that *C. elegans* alter their chemotaxis upon learning by changing the kernels that govern their sensorimotor response.

**Fig 3:**
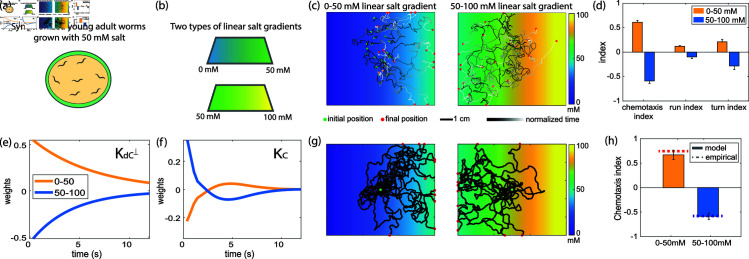
dPAW model captures bidirectional salt chemotaxis. (a) Worms are grown on 50 mM salt environments. (b) Salt chemotaxis are conducted in two types of linear salt gradient plates. (c) Example trajectory for navigation in a 0-50 mM (left) and 50–100 mM (right) linear salt gradient. (d) Summary statistics of measured chemotaxis, reported as in Fig 1d but for salt chemotaxis in two environments. Error bar shows standard error of mean across 10 resampled trajectories from 2–3 salt chemotaxis plates. (e) Kernel $K_{dC^{⊥}}$ and (f) Kernel $K_{C}$ calculated by fitting dPAW to navigation in two linear salt gradients. (g) Example of 11 trajectories simulated for 0–50 mM (left) and 50–100 mM (right) linear salt gradient environments. (h) Chemotaxis index of simulated trajectories with inferred parameters from salt chemotaxis. Error bar shows standard error of mean across 10 repeated simulations, each with 100 trajectories. All data underlying this figure can be found at doi: 10.6084/m9.figshare.24764403 .

### Model also captures bidirectional salt chemotaxis

To gain confidence in dPAW’s ability to extract meaningful sensorimotor kernels and parameters, we also challenged dPAW to capture changes to sensorimotor transformations in a different context, namely context-dependent changes to salt chemotaxis (Fig 3a– 3c). Navigation strategies in a salt environment have been characterized previously—worms navigate towards the concentration they were raised at by employing both biased random walk and weathervaning strategies [18,23,25] (Fig 3d). We repeated a series of experiments previously reported in [23]: animals raised at 50 mM salt concentration were evaluated on linear salt gradients of different concentration regimes (0 to 50 mM, and 50 mM to 100 mM). In each case dPAW was fit to measurements of the animal’s locomotory trajectories. As expected, the dPAW model captured positive and negative salt chemotaxis, depending on the environment the animals were tested on. (The paradigm commonly used for salt chemotaxis differs from that of butanone—for salt the animal’s experience stays the same but the arena varies, while for butanone the experience varies but the arena stays the same.) Reassuredly, simulations from dPAW fitted to salt chemotaxis trajectories nicely agreed with the observed positive and negative chemotaxis indices (Fig 3g and 3h). Sensory kernels had not previously been reported for salt chemotaxis, but one would expect their kernels’ sign to switch depending on whether the worm was in a higher or lower salt concentration landscape. Indeed, dPAW’s inferred salt sensorimotor kernels for both biased random walk ($K_{C}$) and weathervaning ($K_{dC^{⊥}}$) had opposite signs in the high- and low-concentration contexts, as expected (Fig 3e and 3f). The dPAW model’s ability to extract expected parameters and generate trajectories that match observations for salt chemotaxis demonstrates its capacity to generalize across different sensory modalities, environmental landscapes, and concentration levels.

### Decision function governing pirouettes changes upon odor learning

To understand how the animal alters its preference for continuing to weathervane versus interrupting weathervaning with pirouettes during odor learning, we compared the pirouette decision function in Eq 2 inferred from our measurements before and after learning.

**Fig 4:**
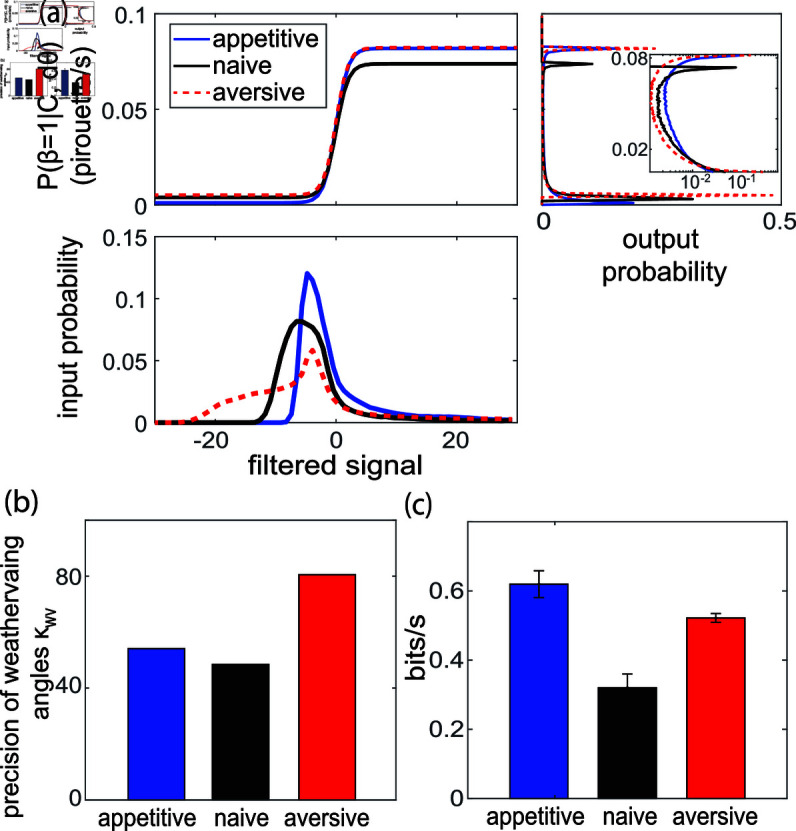
Learning alters pirouette decision and behavioral noise. (a) The top panel shows decision function *P* ( *β* = 1 | *C* , *dθ* )  of three training conditions as a function of filtered signal: $K_{C}\;\cdot \;C\;+\;K_{h}\;\cdot \;|d\theta |$. The distribution of the filtered signal is shown in the bottom panel. The right panel shows the distribution of the output pirouette rate, with inset showing the same distribution in log scale. (b) The precision parameter for weathervaning, $\kappa_{wv}$ across three training conditions. (c) The information rate given fitted model parameters and data. Error bar shows standard error of mean across 7 batches of testing trajectories using cross-validation. All data underlying this figure can be found at doi: 10.6084/m9.figshare.24764403.

We found that learning alters the input-output statistics governing the initiation of a pirouette. This is clear from inspecting the distribution of the filtered signal (Fig 4a, bottom, related to odor concentration and past behavior) that serves as input to the decision function. Appetitive training and aversive training push the tail of the filtered signal probability distribution in opposite directions with respect to naive condition, which changes the statistics of pirouettes. This could reflect either changes to the kernels, or changes to the environment that those animals prefer to explore.

Aversive-trained worms have higher baseline turning rate *m* than appetitive trained worms, as seen in the output probability (Fig 4a, right), which is the result of passing the filtered odor signal (Fig 4a, bottom) through a nonlinear decision function (Eq 2 and Fig 4a, top). Both aversive- and appetitive-trained worms have higher maximum pirouette rate *M* compared to naive worms, reflecting a change to the decision function itself.

It is interesting to note that appetitive (but not aversive) trained animals spend more time with their pirouette probabilities in the most sensitive range of the sigmoid (Fig 4a right, inset), possibly indicating a more efficient strategy for chemotaxis. Capturing these details is one of the ways that dPAW is able to better detect changes in learning.

### Model outperforms other metrics at decoding learned experience

**Fig 5:**
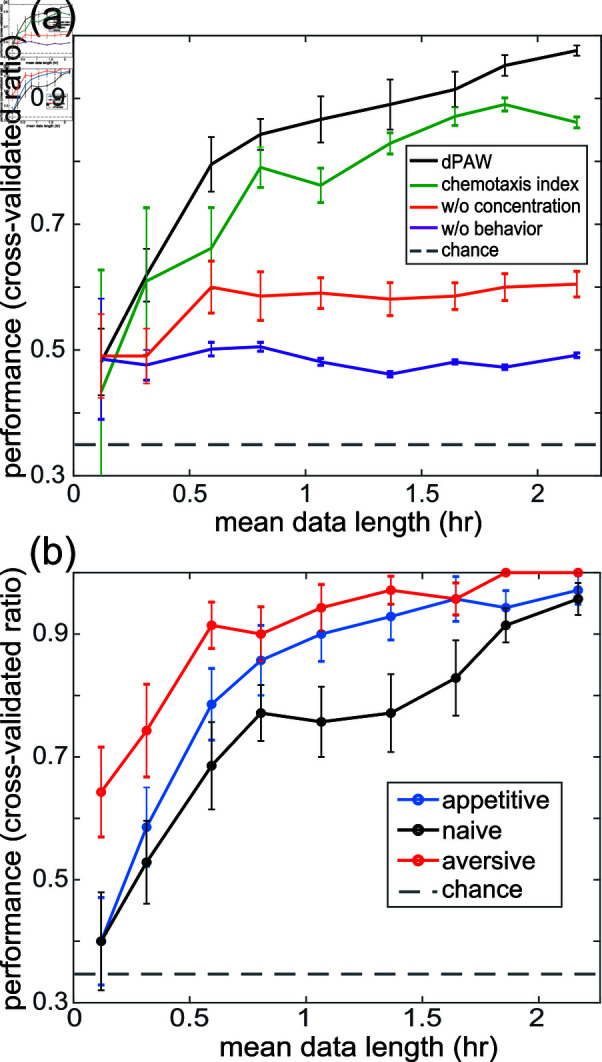
Model-based decoding of learning. (a) Model performance at classifying the prior training of a population of worms (aversive, appetitive or naive) as a function of mean chemotaxis data length. Four models are compared and error bars show standard error of mean across 7-fold cross-validation, each with 10 sampled ensembles across the data length. w/o concentration: model that only captures behavioral statistics and does not account for odor input. w/o behavior: model is given concentration difference along each trajectory and does not have access to behavioral measurements. Chance level, 33%, is shown in grey dashed line. (b) dPAW-based decoding as a function of data length as in (a), but here performance for each training condition is reported separately. All data underlying this figure can be found at doi: 10.6084/m9.figshare.24764403

The changes to sensorimotor processing detected by the dPAW model all provide information about the animal’s past experience. We therefore wondered whether the model could accurately predict the training (aversive, appetitive or naive) that a population of worms had experienced. To test this we inspected dPAW models fit to measured trajectories from each training conditions *γ* ∈ { appetitive , naive , aversive }  corresponding to fitting parameters $\Theta_{\gamma }$ and made maximum likelihood predictions of the training condition given held-out test trajectories: γ^= arg ⁡ max ⁡ γP(dθ→|C→,dC⊥→;Θγ). On held out data, the fitted model correctly predicted training condition with a performance well above 90*%*, significantly above chance levels (Fig 5a), given sufficiently long recordings.

The inferred kernels and decision function within $\Theta_{\gamma }$ better reflect the worm’s learning than either a classic chemotaxis index that captures the fraction of trajectories that go up-gradient (Fig 5a), or the concentration difference along the track Fig G in S1 File. Indeed, on average dPAW always outperforms the chemotaxis index at decoding past training, for any tested amount of finite data. The model’s predictive power is derived from observing both the odor-history experienced by the animal and the corresponding behavior responses. Models supplied with either odor-history or behavior but not both fail to perform as well.

Some training conditions are more challenging for dPAW to decode than others. Naive worms show the lowest predictive performance with finite data when decoding is performed for each training condition separately (Fig 5b). This is consistent with our estimate of lower information exhibited by the trajectories of naive worms than trained worms (Fig 4c). Practically, this means more measurements are required to capture navigation behavior in naive worms than in trained worms.

### The same computations govern natural and optogenetic odor stimuli

We wondered whether the same underlying computations that governed the animal’s response to natural odor stimuli also govern its response to optogenetic-induced sensory stimuli. Optogenetics stimuli are not bound by the same natural statistics that the worm encounters when exploring a physical odor arena. For example, worms in our arena always experience temporally correlated and slowly varying odor responses. This introduces potentially confounding temporal correlations that can be avoided by using optogenetic stimuli [36,38–40]. We therefore investigated the animal’s behavioral response to optogenetic induced odor sensation after learning and adapted our model to incorporate both forms of stimuli.

**Fig 6:**
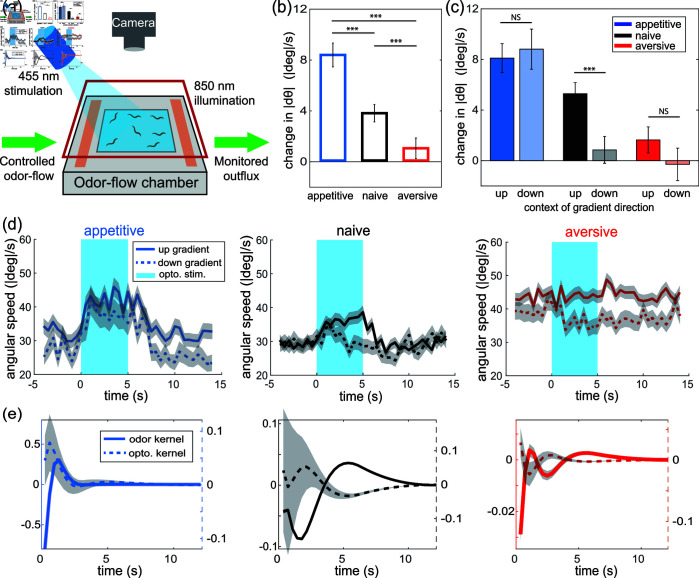
Response to optogenetic induced odor sensation is altered by learning and depends on odor gradient context. (a) Optogenetic stimulation is delivered to animals expressing Channelrhodopsin-2 (ChR2) in AWC^ON^ as they crawl in an odor-flow chamber. (b) Change in the absolute heading  | *dθ* |  upon optogenetic impulse across the three training conditions. This change is defined as the average absolute heading during the 5s impulse minus the value during the 5s preceding the impulse. We record from 4-7 plates per condition. Error bar shows standard error of mean across over 1000 impulses per condition. The results of t-test comparison between training conditions are shown with  ∗ ∗ ∗  for *p* < 0 . 001. (c) Same measurements as (b) upon optogenetic impulse, but with the context of gradient direction. Within each training conditions, measurements are separated into trajectories going up- or down-gradient. The results of t-test comparison between context are shown with  ∗ ∗ ∗  for *p* < 0 . 001 and NS for non-significance. (d) Time-varying average angular speed aligned to the optogenetic stimuli. Three panels show different training conditions. Solid line and dash line indicate up and down gradient trajectories. Gray shading shows standard error of mean across traces. (e) Temporal kernels for odor $K_{C}$ and optogenetic $K_{opto}$ fitted to each training conditions. The grey area shows standard deviation around the estimated kernels. All data underlying this figure can be found at doi: 10.6084/m9.figshare.24764403.

The animal’s behavioral response to optogenetic stimulation was differentially altered by learning (Fig 6; Fig H and Fig I in S1 File). We measured the animal’s absolute change in heading angle  | *dθ* |  in response to optogenetic stimulation of neuron AWC^ON^ expressing ChR2 (Fig 6b). AWC^ON^ is a butanone sensitive neuron known to play an important role in olfactory learning [12,17,36,40]. We measured behavior response to optogenetic stimuli two ways: we delivered pulses of optogenetic stimuli to animals experiencing odor in the arena (Fig 6; Fig H in S1 File), and we delivered time-varying intensity white noise optogenetic stimuli to animals off odor (Fig I in S1 File). In both cases (Fig 6e; Fig I in S1 File) the animal’s turning behavior was more tightly coupled to optogenetic stimuli for appetitive trained animals than naive worms, and less so for aversive trained animals. A change in behavior response is consistent with prior reports [36,40], but note that here we make a more stringent comparison by comparing both appetitive and aversive trained animals against the same naive control condition.

We extended our model so that light intensity contributes to the pirouette probability during navigation:


$$\begin{array}{ccc} P(\beta_{t}=1)=\frac{M-m}{1+\exp(K_{odor}\cdot C_{1:t-1}+K_{opto}\cdot I_{1:t-1})}+m & & \end{array}$$ (5)

where kernels $K_{odor}$ and $K_{opto}$ are weights on vectors of odor concentration $C_{1:t-1}$ and light intensity $I_{1:t-1}$, respectively. Since there is no difference along the perpendicular direction for this five second long spatially uniform optogenetic pulse, this model is simplified with kernels weighting the tangential concentration and neglecting the perpendicular concentration for weathervaning.

Across different training conditions, the optical kernel is close to the mirror image of the odor kernel, most strikingly demonstrated in the naive and aversive conditions (Fig 6e). Therefore the sensorimotor computation inferred from freely moving animals is predictive of the response to external perturbations. The inversion is expected and can partly be explained by the biophysics of the AWC^ON^ neuron: it hyperpolarizes when odor is present and depolarizes when odor is removed. This gives us confidence that our findings about sensorimotor processing derived from natural odor stimuli should be relevant to the larger literature based on more artificial stimulation. We therefore will leverage optogenetics to probe behavioral variability during odor navigation.

### Learning modulates behavioral variability in response to sensory perturbation

We sought to characterize the variability in learned odor-guided navigation, because variability is a known feature of sensory processing in worms [41]. We observe variability across collections of trajectories in learned navigation. For example, the kernel $K_{C}$ that governs the timing of pirouettes varies across subsamples of our data, and seems to vary more for aversive-trained than appetitive-trained or naive animals (Fig F in S1 File).

Surprisingly, we discovered that animals respond to stimuli differently depending on whether they are traveling up or down an odor gradient and that this contributes to variability (Fig 6c and 6d). Naive worms respond more strongly to optogenetic impulses delivered when traveling up an odor gradient, than when traveling down the gradient (Fig 6c). Appetitive trained worms, by contrast, respond consistently to optogenetic impulses regardless of their direction of travel along the odor gradient. Aversive trained worms show weak response to optogenetic impulse regardless of the gradient context. This indicates that learning alters behavioral variability, and that a response to stimuli can be context-dependent along the navigation trajectory.

### Downstream interneurons differentially contribute to learned chemotaxis

**Fig 7:**
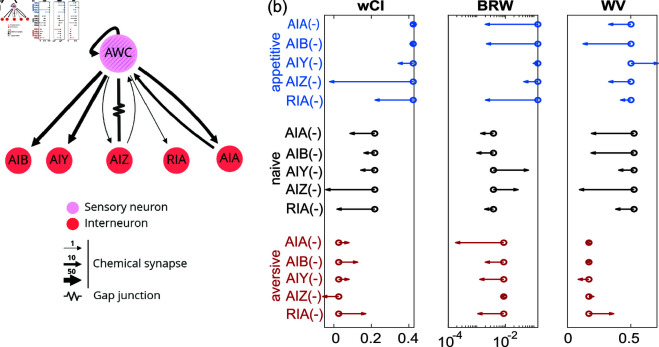
Learned odor navigation in worms with disrupted interneurons. (a) Five interneurons connected to the AWC sensory neuron. Wiring diagram showing synaptic connections, generated from nemanode.org. (b) Weighted chemotaxis index (wCI), biased random walk (BRW), and weathervaning (WV) indices computed from the inferred dPAW for all transgenic strains across three training conditions. BRW and WV correspond to the norm of kernels $K_{C}$ and $K_{dC^{⊥}}$ in dPAW. The circles show value from wild-type N2 worms and arrow shows the modulation in transgenic worms. We record 3-5 plates for each stain and condition, resulting in 200-500 tracks in each measurement. All data underlying this figure can be found at doi: 10.6084/m9.figshare.24764403.

We investigated interneurons that may be involved in implementing learned changes to navigation strategy. We focused on interneurons downstream of the odor-sensing neuron AWC^ON^ because optogenetically induced activity in AWC^ON^ recapitulates aspects of learned navigation (Fig 6b) and AWC^ON^ is known to play an important role in odor sensing [11], navigation [42–44], and butanone learning [3,36]. We selected five interneurons subtypes with direct synaptic inputs (chemical or electrical connections) from AWC^ON^ [10,13]: AIA, AIB, AIZ, AIY, and RIA, several of which are known to be involved in navigation [18,19,23,36,43,45]. For each neuron subtype we compared learned odor-guided navigation in wild type animals to that of transgenic strains for which the neuron subtype was genetically ablated (via miniSOG, Methods Table 1) or down-regulated (via expressing activated potassium channel), as described in methods, Fig 7.

**Table 1:** Table for worm strains.

Strain	Designation	Genotype	Source or reference
Wild-type *C. elegans*	N2	–	CGC
ChR2 expressed in AWC^ON^	AML105	*wtfIs32 [str-2p::ChR2 (H134R)::GFP (50 ng/µl), myo-3p:mCherry (10 ng/µl)]*	this work
AIB(-) activated potassium channel	AML580	*wtfIs491 [inx-1p::twk-18(gf)::mcherry; unc-122p::rfp]*	[32]
AIA(-) miniSOG	IK3240	*njls115[ins-1p::flp,gcy-28dp::frt::tomm-20(N’-55AA)::miniSOG,ges-1p::nls-GFP](IV)*	[19]
AIY(-) miniSOG	IK2962	*njls87[AIYp::tomm-20(N’-55AA)::miniSOG,ges-1p::nls-GFP](IV)*	[19]
AIZ(-) miniSOG	IK3241	*njls116[acc-2p::flp,odr-2(2b)p::frt::tomm-20(N’-55AA)::miniSOG,ges-1p::nls-GFP](IV)*	[19]
RIA(-) miniSOG	IK3289	*njls123[glr-3p::tomm-20(N’-55AA)::miniSOG,ges-1p::nls-GFP](V)*	[19]

Ablating or down-regulating the downstream interneurons had wide-ranging and statistically significant effects on chemotaxis performance and inferred model parameter, including after learning (Fig 7b; Fig J and Fig K in S1 File). Here we characterize chemotaxis performance with a weighted chemotaxis index (wCI) that differs from the chemotaxis index in Fig 1d by more heavily weighting trajectories that experience a large change in odor concentration and de-emphasizing tracks that experience little odor change (described in the methods). To capture changes in inferred model parameters we report a stimuli-normalized magnitude of the kernels $K_{C}$ and $K_{dC^{⊥}}$ which correspond to the extent to which the animal uses the biased random walk (BRW) or weathervaning strategies (WV), respectively, Fig 7b. Normalization is performed by dividing the L2 norm of kernels by the standard deviation of the corresponding sensory signal (described in the methods).

Ablation of neuron subtype AIZ was most severe and eliminated gradient climbing behavior across all three conditions (wCI close to zero), consistent with its reported role in the context of salt chemotaxis [18]. In general, though, neuron subtypes contributed differently to chemotaxis performance, learning, or had different contributions to the inferred kernels corresponding to each navigation strategy. For instance, AIA and AIB defective animals showed little difference between naive and aversive training conditions, but did increase chemotaxis performance after appetitive training. In other words, AIA and AIB are less involved in appetitive behavior but important to maintain naive and aversive behavior. The AIY defective animals show an interesting increase in their use of weathervaning after appetitive training and an increase in their use of biased random walk in naive conditions. This suggests that the recruitment of AIY for different strategies may be learning-dependent. The RIA defective animals have similar chemotaxis indices upon appetitive and aversive learning, suggesting that RIA is involved in fine-tuning the animal’s heading angle in response to learning. Interestingly, RIA defect results in much lower gradient climbing for naive animals. This suggests that, in addition to its role in learning, RIA may also be important for sensory adaptation, including of naive animals, as they perform odor navigation.

In general, we do not observe a simple one-to-one mapping of neuron sub-populations to chemotaxis strategies. This suggests that even though weathervaning and biased random walk strategies are mathematically and behaviorally distinct, they do not segregate cleanly into different neuron types. Instead the neural basis for learning-dependent weathervaning and biased random walks are both distributed across the neural population.

In our investigation, we apply the same model to multiple transgenic strains with different genetic mutations. This has not commonly been done in the past because it can be challenging to find a model that is general enough to accommodate differences across strains. A potential confound can arise if the transgenic strains’ behavior differs from wild-type in such a way as to be poorly captured by the model. And, indeed, we do observe some differences in the locomotory behavior of strains with different neural perturbations, for example their speed distributions differ (Fig Jb in S1 File). Reassuredly, several strands of evidence suggest that the differences dPAW infers in navigation across transgenic strains in Fig 7 reflect differences in navigation strategies, and can not be trivially explained by model mismatch. First, dPAW’s cross-validated fit for transgenic strains is not dramatically different from those of wild-type worms (Fig La in S1 File), suggesting that the model is doing a decent job capturing transgenic strains’ behavior. Second, an analysis of heading distributions further shows reasonable agreement between model and measurements (Fig Lb in S1 File). Finally, the model’s microscopic description of transgenic strains’ behavior does a reasonable job recapitulating the worms’ observed macroscopic chemotaxis index (Fig M in S1 File). When considering all strains across all learning conditions, the model’s predicted chemotaxis index correlated to the measured chemotaxis index ($R^{2}=0.54$). Taken together this suggests that dPAW captures meaningful changes to navigation strategy across strains and that they arise from neuron perturbations. Consequently, this supports the conclusion that learning-dependent navigation strategies are distributed across the neural population.

## Discussion

We combined precise experimental measurements and a novel statistical model, dPAW, to characterize learned olfactory navigation in worms. The results show that (1) navigation strategies are differentially altered by learning, (2) the dPAW model decodes the animal’s past training conditions based on the observed navigation behavior and outperforms a classic chemotaxis metric, (3) behavior is more variable in naive worms and this variability can in part be explained by a context-dependent response to odor, and (4) interneurons downstream from AWC^ON^ contribute to learned navigation strategies in a distributed manner.

To reach these conclusions, two experimental advances were critical. The first was the measurement of navigation in a precisely defined sensory environment enabled by our recently developed odor delivery system [32]. Previous studies had analyzed navigation based on the proximity to a droplet source [3,12,36], but in those experiments the precise odor concentration experienced by the animal was typically not known. With the odor delivery system, we obtained the odor concentration experienced by the animal and therefore were able to fit more comprehensive models of sensorimotor processing.

The second methodological advance is the development of a more robust training protocol to probe differential learning effects (aversive and appetitive) that shows clearer effects when tested in the same odor concentration range. Prior investigations into butanone learning probed different associative learning (appetitive vs. naive, or aversive vs. naive) in different concentration regimes [3,17,36,46], possibly because the behavior effects of aversive learning are known to be more pronounced at lower odor concentrations of butanone while appetitive is known to be more pronounced at higher concentrations of butanone (see for example Fig 1- figure supplement 2 of [12] and results in [47]). But here we sought to directly compare detailed properties of learning, such as kernels, in the same sensory environment. To do so, we changed the training protocol (more repetitions during aversive than appetitive) in order to achieve a greater difference in learning outcomes. This produced large differential training-dependent changes to chemotaxis that were visible even in the same sensory environment (Fig 1).

The dPAW model fitted to measurements provides new insight into how the animal responds to time varying sensory stimuli. In particular, we find that learning alters the temporal kernels for odor input. For instance, appetitive training sharpens the tangential concentration kernel (Fig 2). By contrast, classical approaches have missed this important change because they implicitly have static sensing kernels and *a priori* fix the time change time window to time steps to calculated gradients [16,18,19,23].

Our measurement and model reveals that learning alters not only the sensing mechanism but also the statistics of behavioral noise (Fig 4), which is consistent with recent work showing that starvation and neuromodulation can dramatically alter the statistics of behavior [28,48]. Our finding was possible only because dPAW explicitly includes parameters that control the noise level of the behavioral output, which is another advantage over past work which often excludes noise and relies on predetermined parameters [16,18,49]. Future work is needed to pinpoint the source of behavioral stochasticity, such as noise in the sensory or motor circuits, and its potential functional roles in navigation and exploration [41,50].

A strength of our approach is that it allows us to learn properties of sensorimotor kernels from either artificial optogenetic stimulation or natural odor stimuli. Past work, by contrast, allowed either optogenetic stimulation [36,40] or odor stimuli [34,42], but not both. Optogenetic stimulation has advantages because it can be finely manipulated to deliver rich informative stimuli, for example white noise stimuli to study sensory encoding [51,52]. But there are challenges to connect optogenetic stimuli to natural stimuli experienced during navigation. Our work directly shows that information from one approach is compatible to the other. We applied statistical inference directly to navigational trajectories in the presence of odor and qualitatively recover similar sensorimotor transformations to those we inferred using optogenetic perturbation (Fig 6). This is to our knowledge the first direct comparison between sensorimotor computation during navigation and response upon external perturbation.

An important conclusion from optogenetic perturbations is that the worm’s response variability is altered by learning. We found that naive worms are more variable and that learning reduces variability in behavioral strategies across the population of worms. Surprisingly, we further found that the variation across optogenetic responses in naive worms can in part be explained by the context of that worm’s odor environment, up or down the gradient (Fig 6). Naive worms that travel up-gradient pay more attention to both odor and optogenetic stimuli (Fig 6c; Fig H in S1 File) compared to those that go down-gradient. This is broadly consistent with prior literature describing how the result of associative learning can be heterogeneous across animals or non-stationary in time [53–55]. Measurements in response to optogenetically perturbing AWC^ON^ suggest that the source of such context-dependent behavior might be within or downstream of AWC^ON^. Future work is needed to address the source of such variability in the nervous system, as well as its possible functional role for navigation and searching behavior [1,41].

Of the interneurons we investigated, all five classes contribute to both learned navigation strategies (Fig 7). Importantly, many connections from AWC^ON^ to the first layer of interneurons are shared with salt sensing neurons ASER/L and thermal sensing neuron AFD [18,19,24]. Some past work hypothesize that experience-dependent changes are localized in specific neurons—AIB has been shown to play a role in context-dependent thermal navigation [19]. But other work presents evidence that learning effects can be distributed across many neurons [12,24]. For instance, AIB, AIY, and AIZ all seem to be involved in learned salt chemotaxis strategies [24]. Our findings agree with these results in showing that all three interneurons have non-zero and learning-dependent contributions to both behavioral strategies.

We investigated a selection of key interneurons, but we cannot rule out the role of other neurons that we did not investigate, including conceivably other sensory neurons or modulatory signals [12,36,56,57]. We also studied single neuron-type manipulations one at a time. Future work could explore combinatorial effects by perturbing multiple neuron types at once [6,23]. Finally, we relied on chronic molecular perturbations which risk introducing off-target or compensatory effects to wiring or other neurons during development. Future exploration of neural mechanisms can include more transient perturbations or could be pursued in combination with neural imaging methods.

One puzzling finding is that we sometimes notice changed behavioral strategies but unchanged chemotaxis index. For example, appetitive trained worms with defects in AIB have dramatic reductions in both weathervaning and biased random walk strategies but still show reasonable chemotaxis performance. This may be due to larger model mismatch in transgenic worms with strategies deviating from dPAW. Large variability and less consistent behavioral strategies were also observed in earlier ablation studies [18]. We explore this further in the supplement (Text A in S1 File).

In this work we utilize a controlled odor environment and an innovative model to characterize learned odor navigation in worms. The combined approach of precisely delivered sensory stimuli, behavior quantification and navigational modeling continues to be powerful [9,58,59] and can be generalized to other sensory modalities and species to study adaptive sensory navigation. By identifying the specific features of behavior that are altered by learning, our investigation lays the groundwork for followup neural imaging studies and will guide our search for neural representations of learning [60,61].

## Materials and methods

### Worm strains and preparation

All worms were maintained at 20 C on nematode growth medium (NGM) agar plates seeded with *E. coli* (OP50). We used N2 bristol as wild type worms. A detailed strain list is provided in Table 1. Strain AML105 used for optogenetics experiments was integrated using strain CX14418 from [36] employing UV irradiation and was outcrossed six times before testing. AIB(-) strain is from [32] and AIA(-), AIY(-), AIZ(-) and RIA(-) strains are from [19]. All worm strains used in this work are shown in Table 1.

Before chemotaxis experiments, we bleached and centrifuged batches of worms to synchronize the next generation as described in [60]. L1 synchronized worms were plated to seeded NGM plates on the next day. Experiments were conducted 3 days after seeding, which corresponds to synchronized 1-day-old adult worms. For optogenetic strains, L1 stage worms were plated onto 9 cm NGM agar plates seeded with 1 ml OP50 food with 10 *μ*l all-trans retinal (from 100 mM stock). For interneuron perturbation, miniSOG strains were treated with square wave pulses of 2.16 mW/mm^2^ 450 nm blue light at 1 Hz for 30 minutes at L1 stage and then allowed to recover before testing. The combination of strains and perturbations used in this work are detailed in Table 2.

**Table 2:** Table for experiments and figures.

Experiment	Strain	Odor	Optogenetics	Figures
Odor navigation	N2	*✓*	x	Figs 1 –5; Fig A–G in S1 File.
Simultaneous odor and optogenetics	AML105	*✓*	pulse	Fig 6; Fig H in S1 File.
White noise optogenetics	AML105	x	time-varying intensity white-noise	Fig I in S1 File.
Interneuron disruption	AML580 IK3240 IK3241 IK3289 IK2962	*✓*	x	Fig 7; Fig J–M in S1 File.

### Olfactory learning protocol

The olfactory learning protocol was adapted, with modifications, from previously described protocols [3,12,17,36]. Synchronized young adult worms were removed from food and washed three times with S. Basal solution [11]. For appetitive training, worms were suspended in 10 ml of S. Basal solution on a shaker to starve for 1 hour. After starvation, worms were placed on a 9 cm NGM agar plate with 1 ml of OP50 and 12 *μ*l of pure butanone (2-Butanone, +99%, Extra Pure, Thermo Scientific) dropped on the interior of the lid and sealed with Parafilm. To fixate butanone droplets on the lid, we placed 3 agar plugs on the lid and dropped 4 *μ*l of butanone onto each plug. To conduct aversive training, worms were suspended in 10 ml of S. Basal with 1 *μ*l butanone added. The tube was sealed and placed on the shaker for 1 hour. We found that aversive training was most robust with repetition (Fig A in S1 File), so we interleaved the session by plating the worms back on food for 30 minutes, then repeating the training for three times (Fig 1a). Worms were washed three times with S. Basal solution and centrifuged between each transfer during the training protocol. For naive condition, worms were directly removed from food and washed for three times before testing.

### Odor delivery system and chemotaxis experiments

We used the odor flow delivery system and experimental protocols developed in [32] to measure chemotaxis trajectories after different training conditions. In short, this system incorporates controlled odorized airflow and continuously measures odor concentration along the boundary of an agar plate during animal experiments. As in [32], we calibrated the full array of metal-oxide based sensors with a downstream photo-ionization detector (Fig Ba in S1 File) to characterize the steady-state spatial profile (Fig Bb in S1 File). During animal experiments, we swapped out sensors in the middle of the arena to place in worms on an agar plate and continued to measure the boundary condition to confirm that the odor landscape is controlled and stable across the arena (Fig Bc in S1 File), as in [32] .

For butanone chemotaxis experiments, we used 1.6 *%* agar with salt content matching S. Basal solution in a 10 cm square plate lid. We used 11 mM butanone dissolved in water as the odor reservoir and provided moisturized clean air as the background flow. The background airflow was 400 ml/min and the butanone odor flow was 33–36 ml/min. After the pre-equilibration protocol [32] that brings the agar plate to steady-state in the odor environment, 50–100 worms were placed in the middle of the agar plate and dried with kimwipes. Each chemotaxis sessions was recorded for 30 minutes.

### Behavioral imaging and optical setup

Worm behavior imaging was performed as described in [32]. Briefly, a CMOS camera measured worm behavior at 14 Hz. Worms were illuminated with 850 nm light. Images were captured with a custom written Labview program and analyzed with Matlab scripts. An important difference from [32] is that here we added the ability to deliver optogenetic stimulation. Three 455 nm LEDs (M455L4, Thorlabs) were fixed on top of the flow chamber to deliver stimulation. The intensity was calibrated in the field of view with a photometer, such that 85 *μ*W/mm^2^ intensity light was uniformly delivered. Each pulse lasted for 5 seconds and was delivered every 30 seconds. Here we analyzed 1220 to 3060 pulses delivered to worms treated with retinal in each training condition.

### Chemotaxis assay in linear salt gradients

We conducted salt chemotaxis experiments following established methods [24]. We constructed linear salt gradient in the same 10 cm square plate lids used for odor delivery. Worms grown on standard NGM plates with 50 mM salt concentration were washed off food with M9 solution, then placed in the middle of either the 0–50 mM or 50–100 mM linear gradient. We then imaged behavior in the same odor flow chamber and imaging setup without applying airflow.

### Behavioral analysis and dPAW inference

We tracked the location of the worm’s centroid and fit a centerline to its posture as described in [32]. We removed trajectories that are shorter than 1 minute or have displacement less than 3 mm across the recordings. In addition, trajectories that started above 70*%* of the maximum odor concentration were also removed to prevent double-counting worms that may have already started or traveled up-gradient. This results in 270 to 1,140 animal hours of chemotaxis trajectory per training condition for model fitting.

We characterized chemotaxis performance and strategies with indices in Fig 1d and Fig 3b. The chemotaxis index is $\frac{N_{u}-N_{d}}{N_{u}+N_{d}}$, where $N_{u}$ and $N_{d}$ are the number of tracks going up- and down-gradient, respectively. The run index is $\frac{r_{u}-r_{d}}{r_{u}+r_{d}}$, where $r_{u}$ and $r_{d}$ are run lengths. The turn index is $\frac{p_{u}-p_{d}}{p_{u}+p_{d}}$, where $p_{u}$ and $p_{d}$ are the number of turn events turning up- or down-gradient, respectively.

To analyze the time series of navigation, for each processed navigation trajectory, we computed the displacement vectors every 5 time bins (5/14 seconds) and computed the angle between consecutive vectors to obtain $d\theta_{t}$. We computed the odor concentration it passes through using the two-dimensional odor landscape measured in the flow chamber $C_{t}$. The perpendicular concentration difference $dC^{⊥}$ is calculated with unit vectors that are orthogonal to each displacement vector. We also recorded the length of each displacement vector to form the empirical speed distribution.

We fit dPAW to the ensemble of trajectories by maximizing the penalized log-likelihood:


ΣnNΣtT log ⁡ P(dθn,t+1|Cn,:t,dCn,:t⊥,dθn,:t)−λ|KC|2 (6)

where *N* is the number of trajectories, *T* is the time steps, and *λ* is the regularization hyperparameter determining the strength of the penalty on the squared L2 norm of $K_{C}$. To impose smoothness on kernels, $K_{C}$ is parameterized with 4 raised-cosine basis functions [51], $K_{dC^{⊥}}$ and $K_{h}$ are parameterized with an exponential form. Optimization was performed with constrained optimization in Matlab, where the constrains are positivity of the precision parameters and sigmoid probabilities. All together, there are 13 parameters in the full dPAW model shown in the schematic of Fig 2a, including the nonlinearity and mixture parameters $\left\{M,m,\alpha ,\kappa_{\text{pr}},\kappa_{\text{wv}}\right\}$ shown in Eqs 2–4, 4 coefficients on the bases for $K_{C}$, scale and scale parameters for both $K_{dC^{⊥}}$ and $K_{h}$ exponential kernels. All fitted parameters across conditions and strains are included in the folder: doi: 10.6084/m9.figshare.24764403.

Uncertainty about the inferred parameters are characterized by numerically computing the Hessian of the log-likelihood function around the maximum likelihood estimation. For model-based decoding, we performed 7-fold cross-validation with all measured trajectories. To analyze effects with finite data, we subsampled 10 ensembles of trajectories to test for performance in Fig 5.

To validate the accuracy of maximum likelihood inference for dPAW model parameters, we simulated behavioral time series from dPAW with a fixed set of ground-truth parameters. The model generated simulated trajectories using Gaussian random white noise for concentration time series $C_{t}$ and $dC_{t}^{⊥}$. The time series has 50,000 data points, which is in the same scale of our data length ( ∼  300 animal hours of recording at 5 ∕ 14 Hz sampling frequency). We confirmed that the inference procedure works with simulated data and recovers the ground truth parameters (Fig C in S1 File).

To generate chemotaxis trajectories from inferred parameters shown in Fig 2, we conducted agent-based simulation by measuring concentration in the same odor landscape and drawing angular change $d\theta_{t}$ from dPAW. We simulated two-dimensional navigation trajectories with:


xt+1=xt+vt cos ⁡ (θt) (7)

yt+1=yt+vt sin ⁡ (θt) (8)

$$\begin{array}{ccc} \theta_{t+1} & =\theta_{t}+d\theta_{t} & \end{array}$$ (9)

where $v_{t}$ is speed drawn from Gaussian fit to the empirical distribution.

For information rate (Fig 4) and model comparison (Fig 5), we constructed null models to compare with dPAW. The random walk model has similar statistical structure for behavior but is independent of odor concentration:


$$\begin{array}{ccc} P(d\theta_{t})=P(\beta =1)P_{pr}(d\theta )+P(\beta =0)P_{\text{run}}(d\theta ) & & \end{array}$$ (10)

Note that in this null model, the turning probability is time-independent, so *β* does not have a time subscript *t*. The pirouette behavior has the similar sharp angle as the full model, but now in the null model the weathervaning is changed to “runs” that have zero-mean and do not take perpendicular concentration change into account. Similar to dPAW, the angles are modeled with a von Mises distribution with a concentration parameter. This model was fitted to the same ensemble of trajectories, and the log-likelihood difference between the full dPAW and this null model was normalized by *log* ⁡  ( 2 )  per time to compute the bit rate. We also used this model to compute behavior-only model predictions in Fig 5. The chemotaxis model is formulated with a binomial distribution with expected fraction of tracks going up-gradient $\hat{p}$. The estimation for chemotaxis index is then $2\hat{p}-1$. Lastly, the concentration change model takes $C_{\text{final}}-C_{\text{initial}}$ for all tracks and uses a naive Bayes classifier for prediction.

### Statistical analysis for chemotaxis in transgenic worms

We computed the concentration weighted chemotaxis index (wCI) as follows: $(N_{\text{up}}\Delta C_{\text{up}}-$ $N_{\text{down}}\Delta C_{\text{down}})∕(N_{\text{up}}\Delta C_{\text{up}}\;+\;N_{\text{down}}\Delta C_{\text{down}})$, where *N* is the number of tracks going up- or down-gradient and *ΔC* is computed for every track. We find the maximum and minimum concentration across the full observed odor landscape, then define $\Delta C_{\text{up}}=C_{\text{max}}\;-\;C_{\text{final}}$ for tracks going up-gradient and $\Delta C_{\text{down}}=C_{\text{final}}\;-\;C_{\text{min}}$ for tracks going down-gradient, with ending concentration $C_{\text{final}}$.

To conduct statistical tests between indices in Fig 7, we re-sampled chemotaxis trajectories in each condition (Fig K in S1 File). For the weighted chemotaxis index, we sampled 50 tracks for 20 times to compute the standard deviation from the mean. For the biased random walk strategy, we computed $\left|K_{C}|∕std(C_{t}\right)$ to quantify the weights on concentration *C* and normalized by the standard deviation of the time series itself to compare across strains that experience different concentration inputs. For the weathervaning strategy, we computed $std(K_{dC^{⊥}}*dC_{:t}^{⊥})$ to characterize how much the worm weights and corrects for the heading in response to $dC^{⊥}$. To conduct statistical tests on the behavioral strategies, we sampled 100 times from the posteriors of these kernels, with Gaussian approximation around the maximum likelihood estimate shown in Fig 2 b,c. For each transgenic strain and training condition, we computed the standard deviation of the metrics and conducted t-tests between the transgenic strain and wild type worms (Fig K in S1 File).

### Measuring the behavior-triggered average with optogenetics

For behavior triggered averages (Fig I in S1 File) we delivered time varying LED light stimuli drawn from *N* ( 30 , 30 )  (*μ*W/mm^2^) at 14Hz with 0.5s correlation time and bounded between 0-60 *μ*W/mm^2^. This serves as a white noise stimulus. We computed the behavior triggered average (BTA) for reversals following methods applied to touch sensation in worms [62].

## Supporting information

S1 File Supplementary figures
(PDF)
