# Supplementary material for: Navigation strategies in Caenorhabditis elegans are differentially altered by learning
Source: PLoS Biol. 2025 Mar 21;23(3):e3003005. doi: 10.1371/journal.pbio.3003005 (PMC12135928; doi:10.1371/journal.pbio.3003005)
Supplement: S1 File — Supplementary figures (PDF) [file pbio.3003005.s001.pdf]

Supporting information

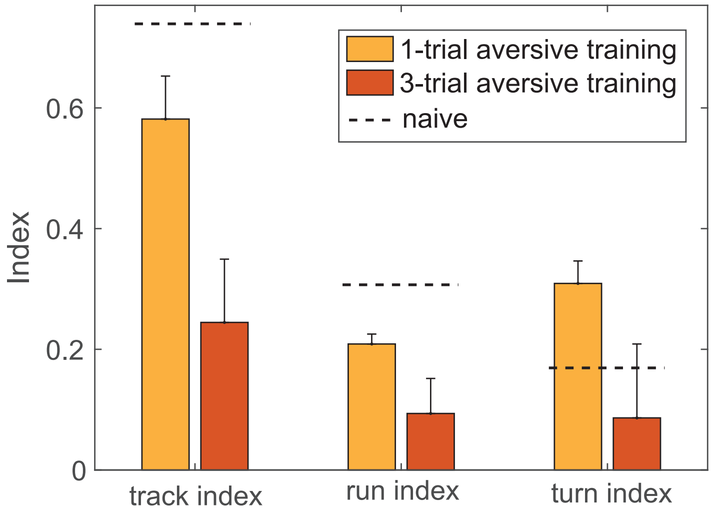

Fig A. Repeated aversive training strengthens the effect of learning. We compared conditions with single-trial of aversive training and with three repetitions. The chemotaxis performance and the usage of two behavioral strategies are computed with the same metrics shown in Fig 1. The values are compared to the mean of naive condition shown in dash line. Error bar shows standard error of mean from 7 plates.

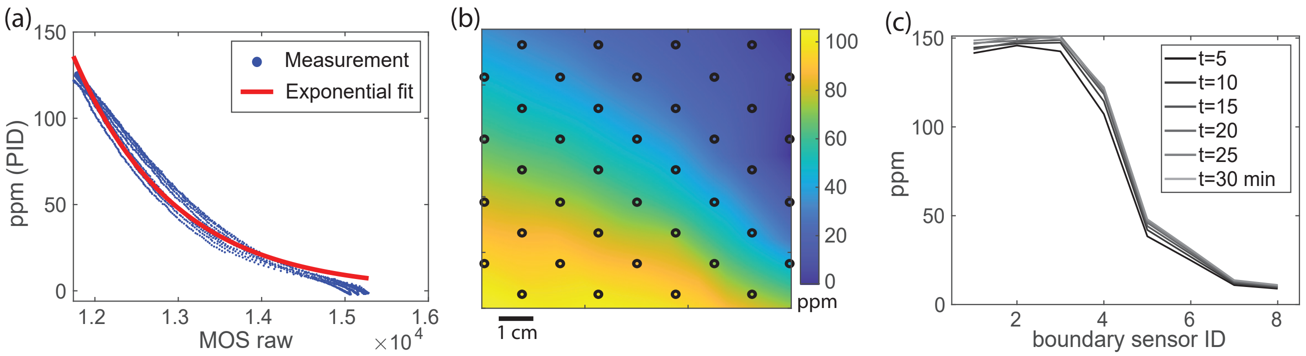

Fig B. Odor landscape experienced by the animal is known, stable across space and time, and calibrated against a photoionization detector. (a) An array of metal-oxide sensors (MOS) is used to monitor odor concentration. The metal-oxide sensor is calibrated against a downstream photoionization detector (PID) to provide parts per million (ppm) of butanone [31]. **(b)** Odor gradient experienced by the animal in a typical experiment, as measured by the full array of sensors indicated in black circles. Inferred odor concentration via interpolation is shown. **(c)** The odor concentration profile along the boundary is stable across the duration of the experiment. Readout from a row of boundary sensors downstream from the odor flow path during animal experiments.

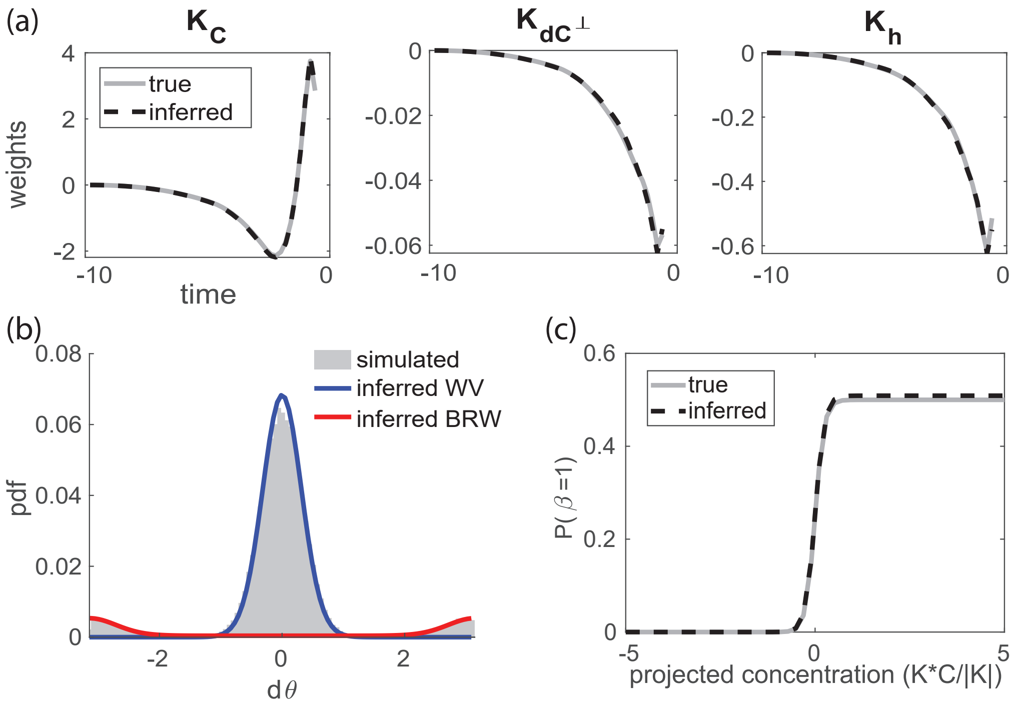

Fig C. The parameters that dPAW learns from the simulation agree with ground truth. Simulated time series is generated with known ground truth parameter, then used to train dPAW. **(a)** Inferred kernels agree with ground truth. **(b)** The $d\theta$ distribution simulated from ground truth parameters overlaid with the inferred densities that come from the same generative model. Densities corresponding to weathervaning (WV) and biased random walk (BRW) strategies are shown in color. **(c)** The sigmoid-like function for pirouette decision as a function of concentration projected onto the normalized kernel K_C is recovered from inference.

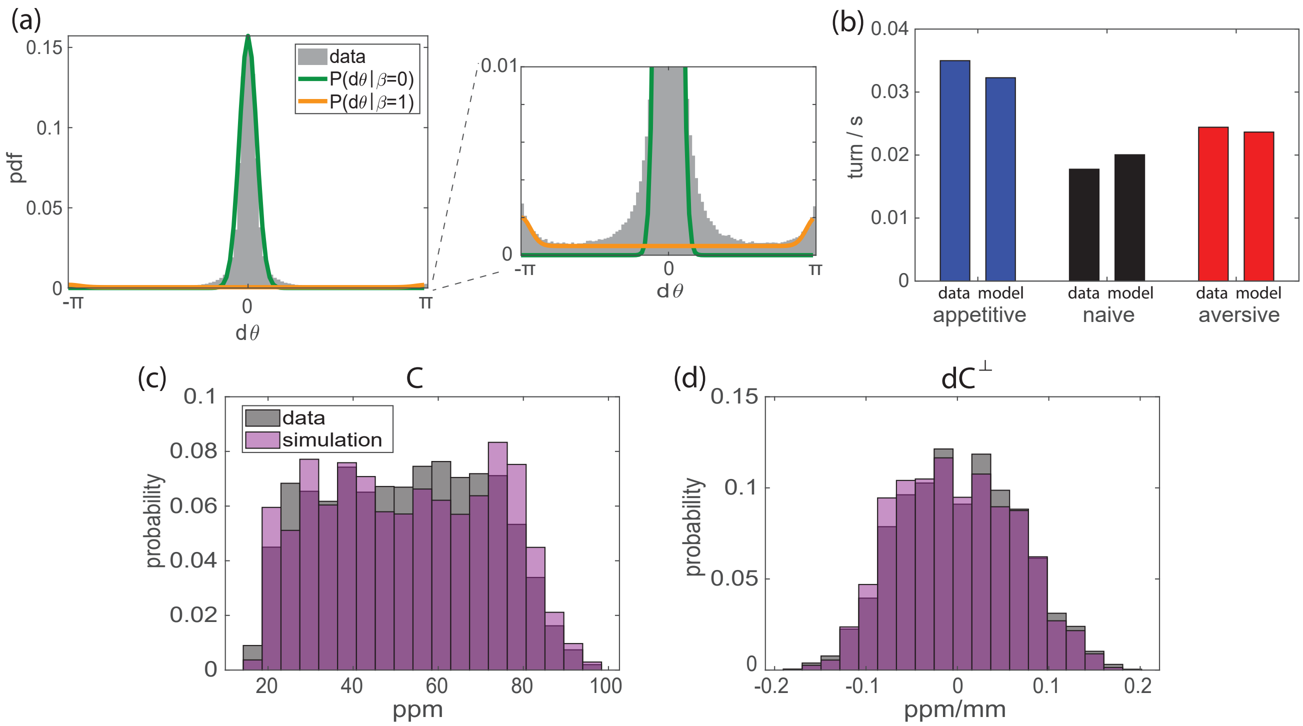

Fig D. The inferred dPAW parameters recapitulate measured sensory and behavioral statistics. (a) The experimentally observed $d\theta$ distribution overlaid with the inferred mixture of two strategies. Zoom-in for a lower probability regime is shown on the right to visualize the pirouette angles ($\beta = 1$). **(b)** The empirical turn rate ($d\theta > 150$ degrees) in data and predicted by the inferred dPAW. The empirical and simulated concentration C distribution **(c)** and perpendicular concentration difference $dC^{\perp}$ **(d)** are shown.

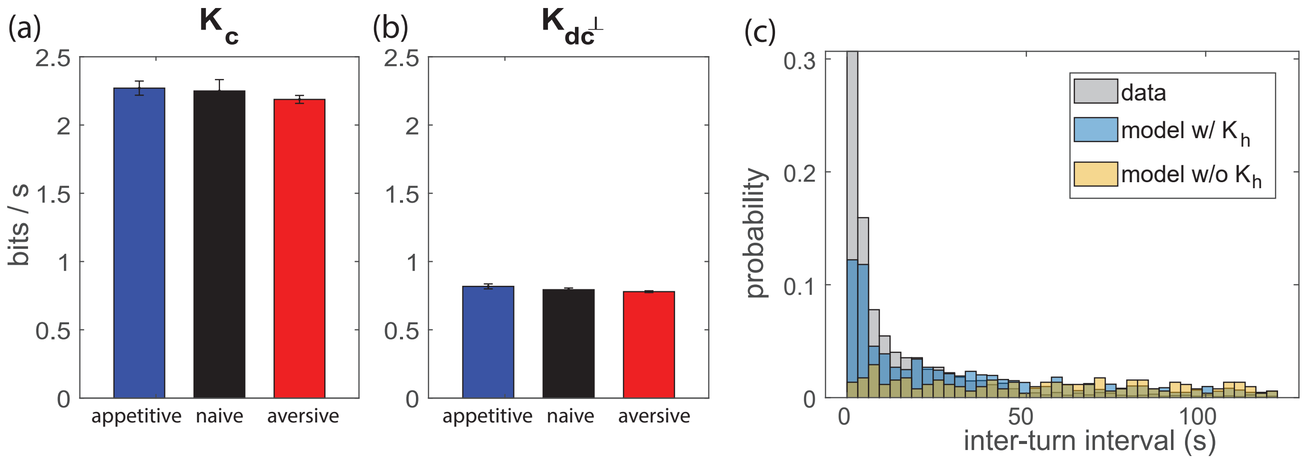

Fig E. The dPAW model has highest performance when it explicitly includes kernels for concentration, perpendicular concentration, and behavior history. Improvement in test log-likelihood (in units of bits / s) due to the inclusion of the specific dPAW model filters is shown. **(a)** Log-likelihood improvement of full dPAW model compared to the model with ablated kernel K_C. Error bar shows standard deviation across 7 fitted ensembles of trajectories. **(b)** Same as (a) but with ablated kernel $K_{dC^{\perp}}$. **(c)** The distribution of inter-turn-intervals observed from data, compared to trajectories simulated from the inferred dPAW with or without kernel K_h. It has been reported that worms produce sharp turns in bouts with specific time scales, and the original definition of a pirouette included events defined by multiple sharp turns close together in time [16]. The results show that the dPAW model can generate tracks that have similar kinetics with the history kernel and without explicitly modeling state-transitions.

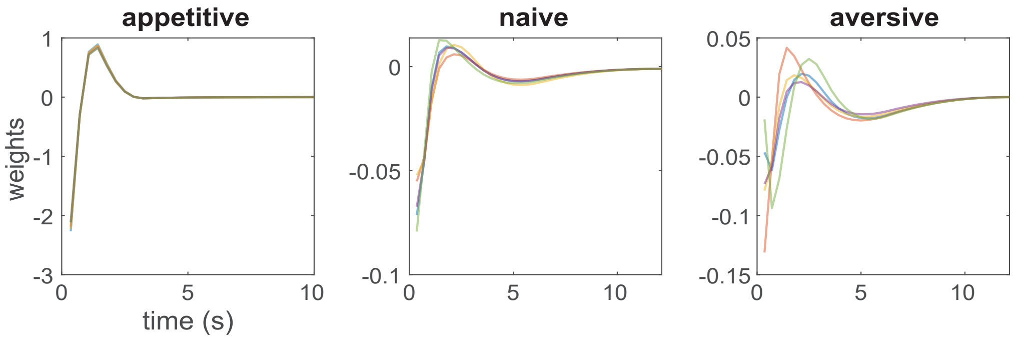

Fig F. Variability of the inferred kernels K_C across 5 datasets sub-sampled (without replacement) from the full dataset, for each of the three training conditions. The inferred kernels K_C for each subsample are shown in different color coded lines. The estimated kernels were highly consistent in appetitive condition, but more variable in naive and aversive conditions.

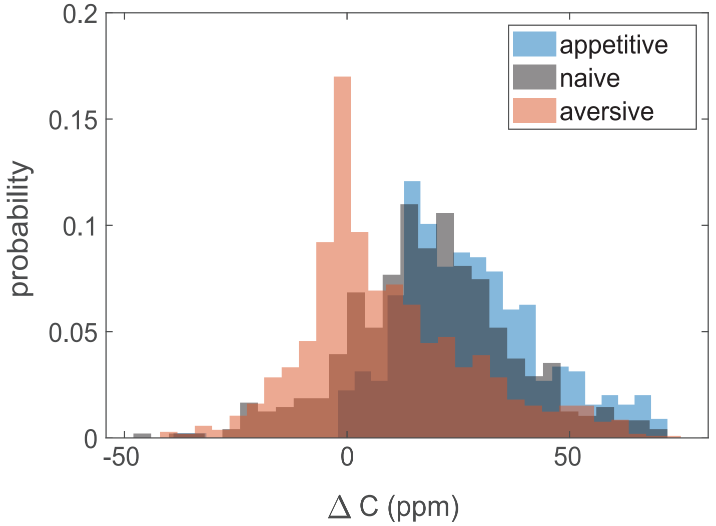

Fig G. Histogram of the odor concentration change ΔC along all trajectories observed after three different training conditions. The aversive condition has a qualitative shift away from appetitive and naive conditions. However, as quantified in Fig 5a, concentration alone has lower prediction power compared to the full dPAW model.

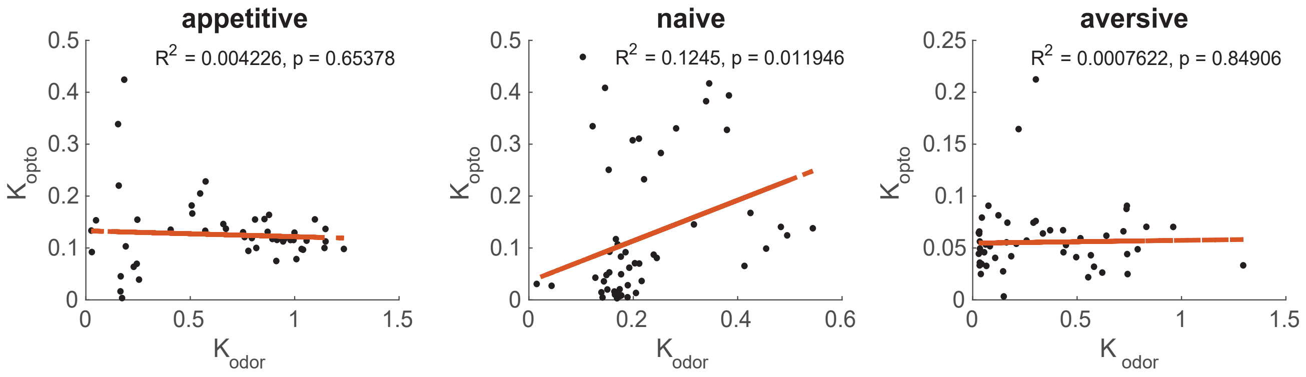

Fig H. Optogenetic kernel weights co-vary with odor kernel weights in naive but not learned conditions. To examine the relation between odor and optogenetic sensorimotor processing, we sub-sampled trajectories recorded from chemotaxis with optogenetic perturbation. For each sub-sampled navigation trajectories, we fit the statistical model for pirouette probability (equation 5) and computed the norm of kernels K_{opto} and K_{odor}. We re-sampled ensemble of 100 trajectories from 300-1000 trajectories across three learning conditions. The scatter plots compare the norm of kernels K_{opto} and K_{odor} inferred from sub-sampled datasets. The scattered samples show weak but significant correlation in naive condition. This means that subset of trajectories that vary and have less weight on odor signal would also have less optogenetic response. In contrast, aversive worms have clustered small weight on odor and is uncorrelated to the optogenetic input. Together, the sub-sampling result is consistent with the observation in Fig 6 and supports the finding that tracks going down-gradient (less weight on odor input) also respond less to optogenetic input.

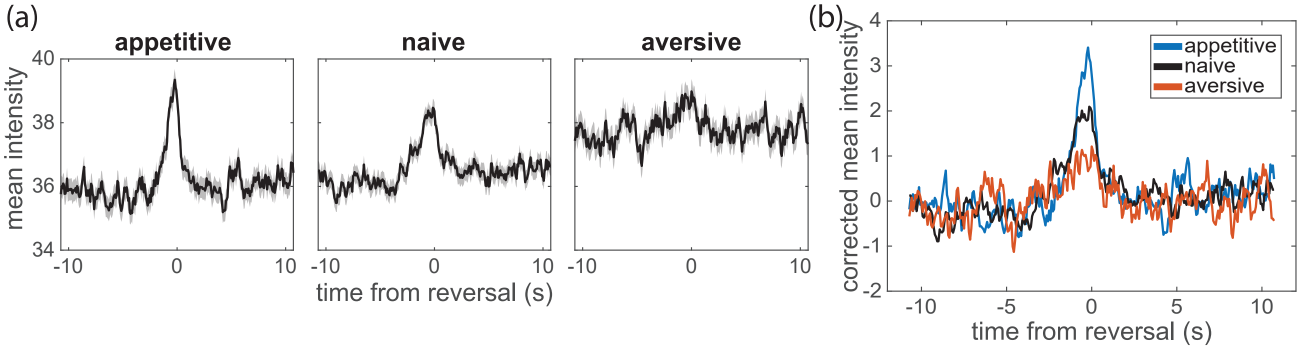

Fig I. Optogenetic kernels extracted from white-noise optogenetic stimulation measurements. Changes to these optogenetic kernels mimic changes to the odor kernels that were observed in odor landscape. **(a)** Behavioral triggered average (BTA) for reversal computed from Gaussian white noise stimuli after three training conditions. The shaded area shows standard error of mean around the mean intensity (μW/mm^2). **(b)** Comparing BTA from (a) after baseline subtraction for three training conditions.

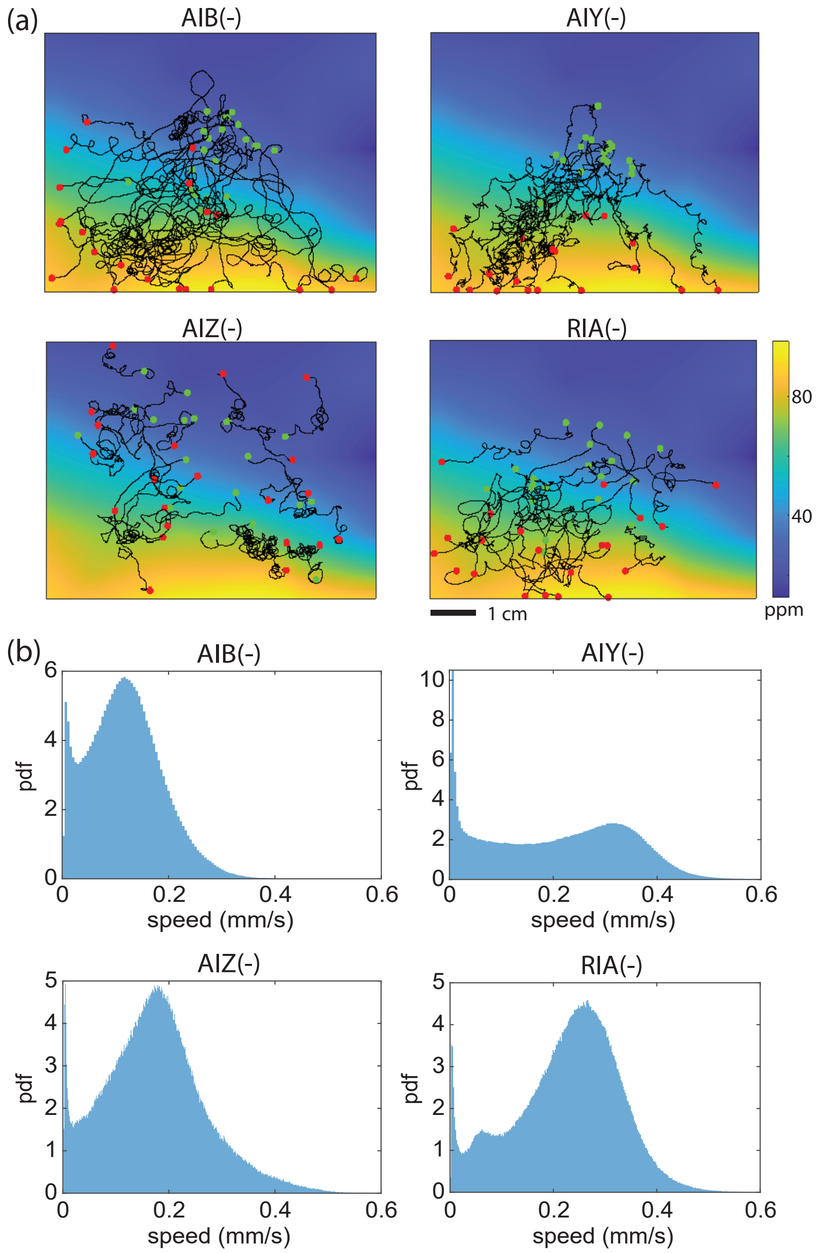

Fig J. Disruptions to different interneurons result in qualitatively different locomotion trajectories. (a) Trajectories from worms after appetitive training for disruptions to four different classes of interneurons are shown. Green dots and red dots indicate the starting point and ending point of each track, respectively. **(b)** Same measurements as (a) but for the speed distribution during chemotaxis across strains.

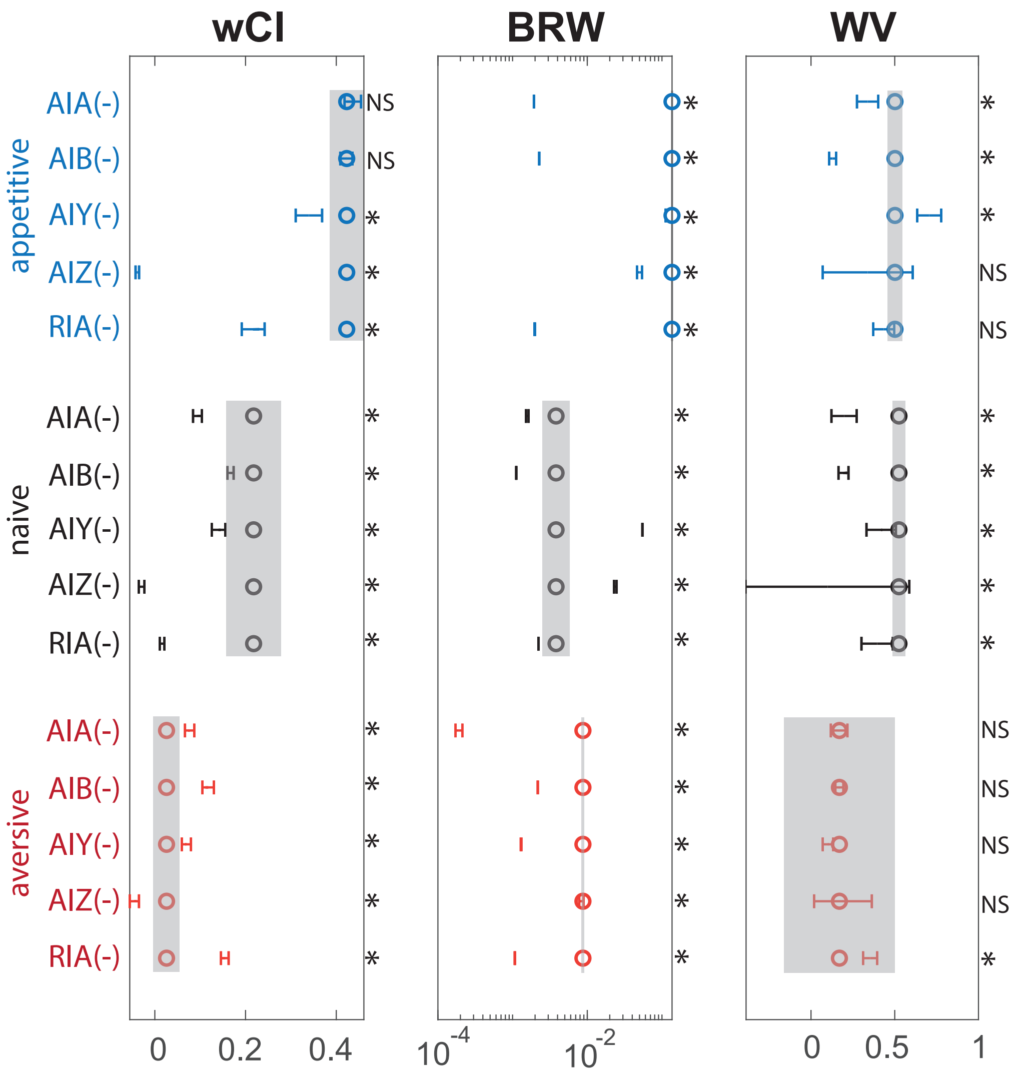

Fig K. Statistical tests for learned chemotaxis and behavioral strategies across worm strains with disrupted interneurons. For wild-type worms, the mean values of weighted chemotaxis index (wCI), biased random walk (BRW), and weathervaning (WV), are shown in circle in each panel. The standard deviations of these values are indicated with the shaded area. Three different learning experiences are shown in color. For different transgenic strains across different learning experiences, the mean and standard deviation are plotted for comparison to wild-type. The mean and standard deviation for wCI are computed from 20 re-sampled trajectories. The mean and standard deviation for BRW and WV are computed from 100 samples from the maximum likelihood estimation of kernels and the Hessian around it. We conduct t-test between the samples from measurements in transgenic strains and wild-type measurements and indicate the result on the right side of each panel ($*$ for $p < 0.01$ or non-significance, NS; using Bonferroni correction for multiple comparisons test).

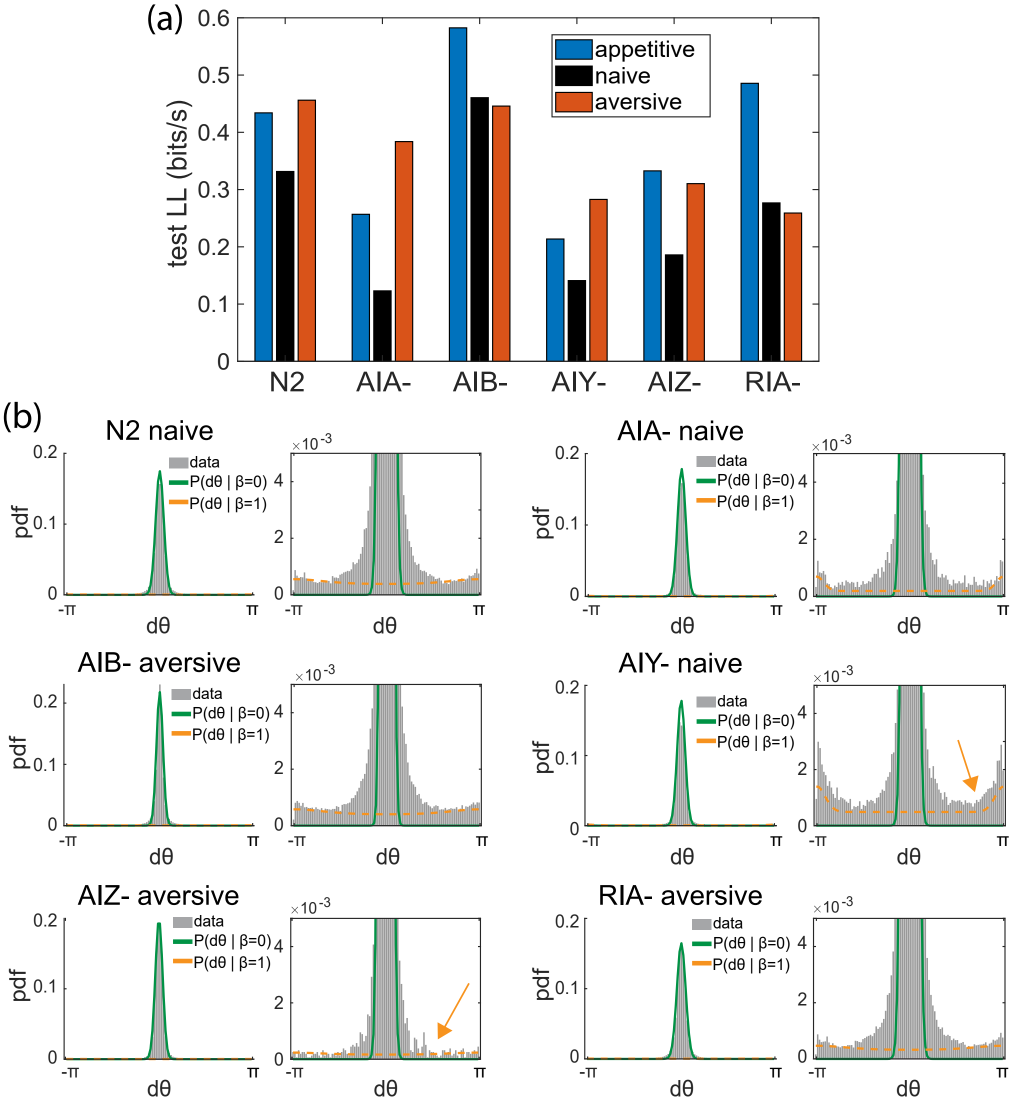

Fig L. The dPAW model fits to different transgenic strains. (a) Test log-likelihood (testLL) across all worm strains and learning conditions. The testLL value is computed for held-out test dataset and subtracting the model fit to another null model with ablated sensory kernels. The fact that testLL for all strains are positive in the testing set means that the fitted parameters do generalize across data, without significant overfitting. **(b)** Examples of heading densities and the model predictions across strains. We notice that for certain strains, such as AIY and AIZ strains (orange arrows), may have poorer fits due to the under-estimated turning rate. However, the overall fits demonstrated the flexibility of dPAW to capture a variety of behavioral features, with different pirouette rates and shapes of the broad angle distribution. Together, the cross-validation analysis provides validation and confidence for transgenic strain analysis in Fig 7.

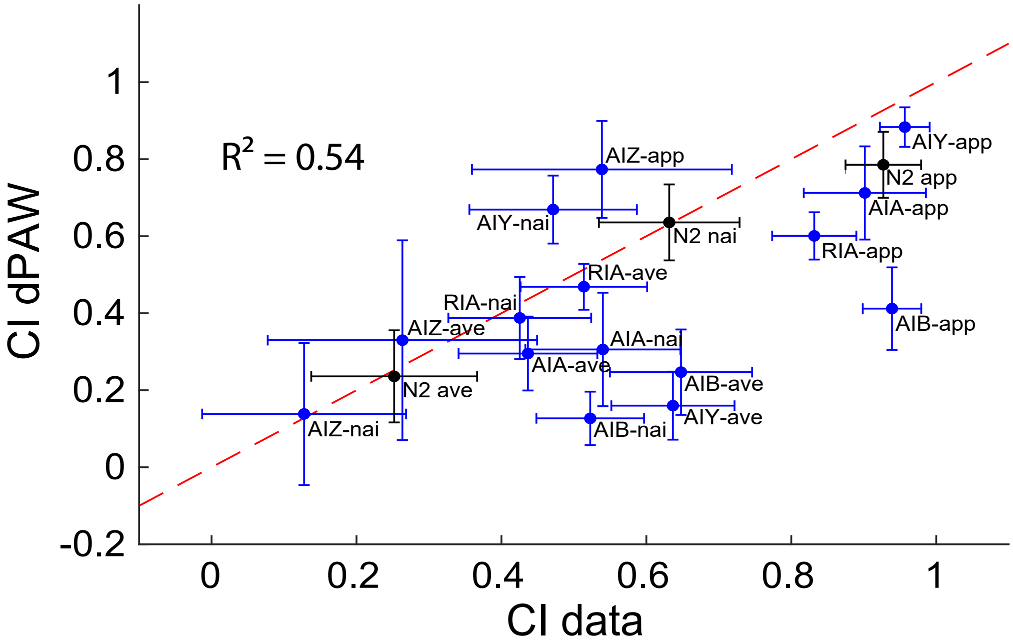

Fig M. Generating chemotaxis with dPAW fitted across strains and learning conditions. We investigated chemotaxis index (CI) from trajectories simulated from dPAW fitted across all worm strains and found that a good portion of them produce match to experimental data. Appetitive, naive, and aversive conditions are abbreviated as app, nai, and ave, respectively. Red dashed line indicates the diagonal when the dPAW model and experimental data have the same CI. Error bars show standard deviation from 50 repeated samples of tracks from each condition. The R^2 value indicates correlation across all strains. Specifically, strains such as AIB- and AIY-aversive showed larger deviation from the correct chemotaxis indices through dPAW simulations. While this indicates that dPAW lacks the flexibility to capture strategies in these transgenic strains, we believe that it does not necessarily devalue the interpretation of the fitted parameters. In machine learning terms, there may be a larger model mismatch for dPAW in these strains and learning conditions. Future work is required to develop a more general model for all behavioral repertoire across strains.

Text A. Supplementary text for discussion on limitations and future work. With the dynamic Pirouette and Weathervaning (dPAW) model fitted to experimental data, we confirmed that the inferred densities for head angles $d\theta$ match the observed distribution (Fig Da). Two mixtures of von Mises distributions in dPAW can approximate the features observed from experiments. We find the agreement to be overall very good. But we noticed that where there is mismatch, it is largest in a range of intermediate angles. It is possible that this mismatch could be explained by more subtle aspects of locomotion that have previously been proposed to be relevant for navigation in worms [19], such as omega-shape and J-shape turns. Future work could explore whether adding these additional features to dPAW improves agreement.

Another main assumption in dPAW, as well as other chemotaxis characterization in previous work [16,18], is that the behavioral responses are stationary through time. It is possible that worms can respond differently in response to different local concentration gradients. Future work is needed to characterize non-stationary behavioral dynamics during chemotaxis.

When investigating the relationship between the biased random walk (BRW) indices, the weathervaning (WV) indices and the weighted chemotaxis index (wCI) (Fig 7b), we were surprised and initially puzzled to find that changes to the indices did not always correspond to a change in the chemotaxis performance. In other words, chemotaxis performance, at least by this measure, isn't trivially the sum of the BRW and WV indices. For instance, AIA(-) and AIB(-) worms are appetitive trained have the same weighted CI as wild-type, but their BRW and WV index are significantly altered. Previous work has also observed a similar mismatch between indices for behavioral strategies and chemotaxis performance [18].

Based on this observation, we speculate that it is possible that ablated or disrupted worms can adaptively employ different strategies, including those not captured by the BRW or WV indices, to reach similar chemotaxis performance. For instance, we qualitatively observed that AIB(-) worms have less sharp turns and have curving trajectories in space, but still end up moving up-gradient (Fig J), suggesting a possibly alternate strategy. In contrast, AIY(-) worms have high rate of sharp turns and move up-gradient with non-smooth trajectories. Future work with models that have alternative strategies or more hierarchical structures is needed to characterize the complexity of these chemotaxis patterns.
